# Diagnostic and Prognostic Performance of Liquid Biopsy-Derived Exosomal MicroRNAs in Thyroid Cancer Patients: A Systematic Review and Meta-Analysis

**DOI:** 10.3390/cancers13174295

**Published:** 2021-08-26

**Authors:** Eman A. Toraih, Rami M. Elshazli, Lily N. Trinh, Mohammad H. Hussein, Abdallah A. Attia, Emmanuelle M. L. Ruiz, Mourad Zerfaoui, Manal S. Fawzy, Emad Kandil

**Affiliations:** 1 Department of Surgery, School of Medicine, Tulane University, New Orleans, LA 70112, USA; mhussein1@tulane.edu (M.H.H.); aattia@tulane.edu (A.A.A.); remmanuelle@tulane.edu (E.M.L.R.); mzerfaoui@tulane.edu (M.Z.); 2 Genetics Unit, Department of Histology and Cell Biology, Suez Canal University, Ismailia 41522, Egypt; 3 Department of Biochemistry and Molecular Genetics, Faculty of Physical Therapy, Horus University-Egypt, New Damietta 34517, Egypt; Relshazly@horus.edu.eg; 4 School of Medicine, Tulane University, New Orleans, LA 70112, USA; ltrinh1@tulane.edu; 5 Division of Thyroid and Parathyroid Endocrine Surgery, Department of Otolaryngology-Head and Neck Surgery, Massachusetts Eye and Ear Infirmary, Harvard Medical School, Boston, MA 02114, USA; 6 Department of Medical Biochemistry and Molecular Biology, Faculty of Medicine, Suez Canal University, Ismailia 41522, Egypt; manal2_khashana@ymail.com; 7 Department of Biochemistry, Faculty of Medicine, Northern Border University, Arar 1321, Saudi Arabia

**Keywords:** thyroid cancer, exosomal microRNAs, miRNA, liquid biopsy, meta-analysis

## Abstract

**Simple Summary:**

Circulatory tumor-derived exosomal miRNAs play key roles in cancer development and progression. Studies have shown that serum and plasma miRNAs have the potential to be promising biomarkers for cancer diagnosis. This meta-analysis aimed to assess the diagnostic and prognostic performance of exosomal miRNAs in thyroid cancer. Our study analysis included 12 articles. We found that specific exosomal miRNAs found in blood provide high diagnostic value with high sensitivity and specificity. Furthermore, certain panels of exosomal microRNAs showed remarkable diagnostic value. The best discriminative ability to differentiate between cancer and non-cancer individuals were for miR-146b-5p + miR-223-5p + miR-182-5p. The novel and non-invasive use of miRNAs to diagnose TC can significantly improve patient outcomes by preventing the burden of unnecessary surgery and providing prognosis information on thyroid cancer.

**Abstract:**

Circulatory tumor-derived exosomal microRNAs (miRNAs) play key roles in cancer development/progression. We aimed to assess the diagnostic/prognostic value of circulating exosomal miRNA in thyroid cancer (TC). A search in PubMed, Scopus, Web of Science, and Science Direct up to 22 May 2021 was performed. The true/false positive (TP/FP) and true/false negative (TN/FN) rates were extracted from each eligible study to obtain the pooled sensitivity, specificity, positive/negative likelihood ratios (PLR/NLR), diagnostic odds ratio (DOR), and their 95% confidence intervals (95%CIs). The meta-analysis included 12 articles consisting of 1164 Asian patients and 540 controls. All miRNAs were quantified using qRT-PCR assays. The pooled sensitivity was 82% (95%CI = 77–86%), pooled specificity was 76% (95%CI = 71–80%), and pooled DOR was 13.6 (95%CI = 8.8–21.8). The best biomarkers with high sensitivity were miR-16-2-3p (94%), miR-223-5p (91%), miR-130a-3p (90%), and miR182-5p (94%). Similarly, they showed high specificity, in addition to miR-34c-5p. Six panels of two to four exosomal miRNAs showed higher diagnostic values with an area under the curve (AUC) ranging from 0.906 to 0.981. The best discriminative ability to differentiate between cancer and non-cancer individuals was observed for miR-146b-5p + miR-223-5p + miR-182-5p (AUC = 0.981, sensitivity = 93.8% (84.9–98.3), specificity = 92.9% (76.5–99.1)). In conclusion, the expression levels of exosomal miRNAs could predict TC.

## 1. Introduction

Thyroid cancer (TC) growth is one of the most common malignant tumors in the endocrine system [1]. The incidence of thyroid cancer increases with an annual rate of 5.4% in men and 4.6% in women [2]. Ultrasound imaging, positron emission tomography-computed tomography (PET-CT), and fine-needle aspiration biopsy (FNA) are widely conducted to determine the properties of the masses and confirm the diagnosis [3,4]. However, these strategies have their limitations of being expensive, invasive, time-consuming, or overly dependent on the medical staff’s precise instruments and technical levels [5,6]. Financial distress and adverse financial events were common among thyroid cancer survivors and were associated with a more inferior health-related quality of life [7]. Moreover, about 10–40% of FNA cytology analysis cannot confirm the malignancy, and many patients undergo unnecessary thyroidectomy for benign lesions [8]. Therefore, novel non-invasive methods for diagnosis of TC have the potential to improve patient outcomes significantly.

MicroRNAs (miRNAs) are a group of small non-coding RNA molecules with a length of 21–23 nucleotides [9]. They regulate the expression of multiple protein-coding genes at the post-transcriptional level and are implicated in controlling signaling circuits within a cell [10]. Studies showed that miRNAs are dysregulated in human malignancies and play an essential role in the evolution and progression of cancer [11,12]. Furthermore, functional studies show that miRNAs affect TC cell proliferation, migration, and invasion [12,13]. In addition, studies showed that several of these miRNAs are related to prognosis and can serve as diagnostic markers [14].

Exosomes are vesicles with a size of 30–150 nm in diameter. They are essential for cells to communicate with neighboring cells or with distant cells [15]. All exosomes hold surface molecules that help them to target the recipient cells. Once attached to the recipient cells, the exosomes fuse with the cells’ membranes to release their cargo into target cells, thereby changing the physiological state of the recipient cells. In addition to intra-cellular regulatory functioning, miRNA can be secreted by cells into interstitial spaces to shuttle the regulatory signal to neighboring and distant cells. Detection of tumor-derived miRNA in various bodily fluids may also be helpful for both early cancer diagnostic and therapeutic management [16]. Exosomal miRNAs are more stable than free miRNAs in circulation as they are more resistant to the proteolytic activity of ribonucleases [17,18]. Therefore, exosomal miRNA can serve as potential diagnostic and prognostic biomarkers. Previous studies suggest promising results of exosomal miRNA in diagnosing several human cancers, such as glioma and breast cancer [19,20]. In addition, studies report that expression levels of exosomal miRNAs in plasma of patients with TC were significantly different, suggesting that exosomal miRNAs have great potential to be biomarkers for TC [21]. For example, plasma exosomal miR-146b-5p and miR-222-3p have been suggested as potential biomarkers for lymph node metastasis (LNM) in papillary TC (PTC) [20].

While prior studies have evaluated the novel use of exosomal miRNA in various cancers, the global profiling of exosomal miRNAs from plasma or serum of patients with TC has not been widely investigated. This systematic review and meta-analysis aimed to evaluate liquid biopsy-derived exosomal miRNAs from serum and plasma as diagnostic and prognostic tools in TC.

## 2. Materials and Methods

### 2.1. Literature Search Strategy

The design of this current meta-analysis and systematic review was executed utilizing the preferred reporting items for systematic reviews and meta-analyses (PRISMA) protocols [22]. A systemic search was performed using the following search engines: PubMed, Scopus, Web of Science, and Science Direct up to 20 May 2021. The inclusion criteria set was adopted utilizing a combination of keywords involving (“exosomal miRNAs”, “exosomal miRNAs”, “exosomal miRs”, “exosome miRNAs” or “exomiRs) and (“thyroid cancer”, “thyroid carcinoma”, “thyroid neoplasm” or “thyroid tumor”) and (“prognosis” or “survival”). Additionally, the bibliography lists were screened manually to identify other reports.

### 2.2. Inclusion and Exclusion Criteria

The selected inclusion criteria were as follows: (1) studies involved human samples in which the expression level of exosomal miRNAs was in serum, plasma, or blood of thyroid cancer patients; (2) any study type: observational or diagnostic accuracy studies; (3) detection method for miRNA profiling is clearly defined in the article; (4) proven diagnosis of non-medullary thyroid tumor by histopathology; (5) comparisons of cancer and normal subjects or cancer and benign disease as nodular goiter or thyroid adenoma; (6) reported at least one prognostic outcome as tumor size, lymph node metastasis, extrathyroidal extension, recurrence, overall survival (OS), disease-free survival (DFS), disease-specific survival (DSS), or progression-free survival (PFS); (7) measures of true positive (TP), true negative (TN), false positive (FP), and false negative (FN) could be extracted or estimated from reported sensitivity and specificity; (8) reported odd ratio (OR), relative risk (RR), hazard ratio (HR), or an area under the curve (AUC) and their 95% confidence intervals (CI) for the predictive ability of exosomal miRNAs expression to predict poor prognosis; and (9) no limitations for sex, age, or geographical distribution.

Exclusion criteria were as follows: (1) The editorial materials, literature reviews, letters, or meetings; (2) repeated research publications/duplication; (3) the expression of miRNAs was detected within tumor tissues or other body fluids; (4) literature with insufficient and overlap data; (5) in vitro and in vivo studies; and (6) non-English articles.

### 2.3. Quality Assessment

The quality evaluation was conducted by (ASA) to assess the degree of quality of nonrandomized studies in meta-analyses based on the Newcastle–Ottawa quality assessment scale (NOS). This assessment was developed based on the star system that applied three levels of judgment involving (a) the selection of the study group, (b) the comparability within the groups, and (c) the ascertainment of the outcome for the studies. The NOS scores varied from 0 to 9. Six points or more were deemed as high quality [23]. Furthermore, evaluation of the enrolled studies according to the guidelines on experimental methods and minimal information for studies of extracellular vesicles (MISEV) based on three main domains: “(i) EV isolation/purification, (ii) EV characterization, and (iii) EV functional studies” [24] has been applied. One point has been assigned to each criterion, which should yield a total score of 10 if fulfilled (Table S1).

### 2.4. Data Extraction

Three investigators (ET, RE, and MH) independently extracted the information and data from all eligible studies. The information of each study was abstracted using a pre-designed form: the first author, the year of publication, the research country, the subtype of thyroid cancer, the overall number of patients and controls, method of profiling method, and demographics of the patients. Prognostic outcomes, including survival, were also reported. The AUC, sensitivity, specificity, and fold change were collected. The disagreements between the three investigators were settled by discussion until an agreement was reached with the fourth investigator (MSF).

### 2.5. Statistical Analysis

None of the articles reported directly diagnostic accuracy measures (TP, TN, FP, and FN); therefore, they were calculated using MedCalc from sensitivity, specificity, and area under the curve. Meta-Disc v1.4 and RStudio were used for statistical analysis and generating random forests and other plots. Pooled sensitivity, specificity, likelihood ratios, and diagnostic odds ratio were estimated. The outputs are presented numerically and graphically as forest plots. Pooled estimates are provided with their respective confidence intervals. The DerSimonian Laird method was used to estimate an overall diagnostic odds ratio. The heterogeneity was tested by I2 values; if I2 < 50%, a fixed-effects model was used; otherwise, the random-effects model was applied. Finally, a hierarchical summary receiver operator characteristic (sROC) was employed to estimate AUC and the Q* index as a summary measure of global accuracy of miRNA testing. AUC > 0.75 represented high diagnostic efficacy. Spearman correlation analysis was performed to trace the source of heterogeneity due to the threshold effect: *p* > 0.05 would indicate the absence of the threshold effect, and thus, all miRNAs could be combined. A subgroup analysis was conducted according to the type of comparison. Meta-regression was implemented using a generalization of the Littenberg and Moses Linear model weighted by the inverse of the variance to explore the impact of the type of comparison on heterogeneity.

## 3. Results

### 3.1. Characteristics of Included Studies

**Figure 1:**
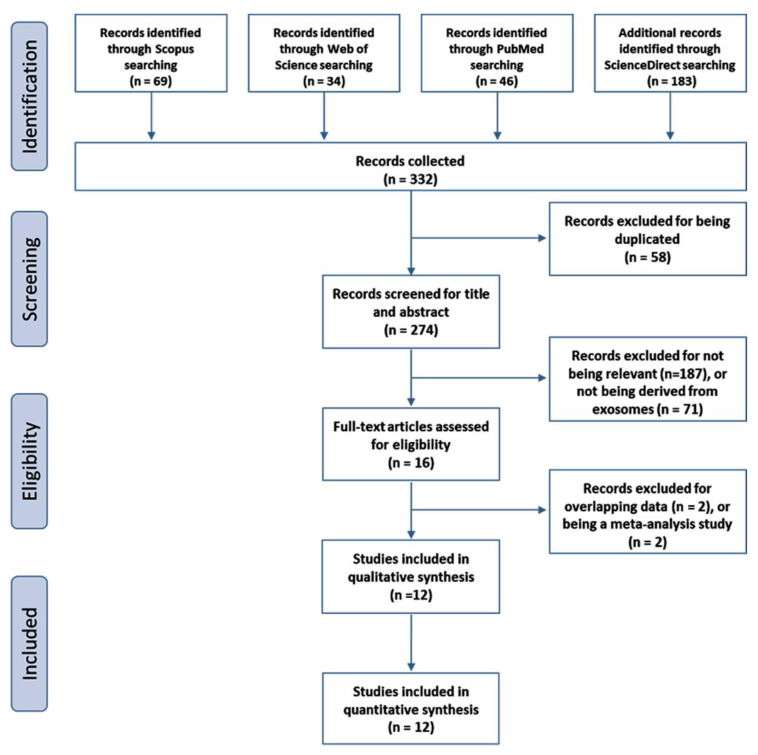
Flow chart describing search strategy. Workflow of study selection according to the PRISMA guidelines [22].

A total of 332 articles were initially screened, and 274 articles remained after 58 duplicates were eliminated. The literature was then screened according to title and abstract, and 187 more studies were excluded for being irrelevant and an additional 71 articles for not being derived from circulatory exosomes. The full text of the remaining 16 studies was reviewed in-depth, and 4 additional studies were excluded. Finally, the remaining 12 articles, including 1164 patients and 540 controls, met the inclusion criteria (Figure 1). Apart from a single study [25], all articles were conducted in China [21,26,27,28,29,30,31,32,33,34,35]. They were published between 2016 and 2021. The sample size ranged from 10 to 491 patients per study (Table 1). Quantitative Real-Time Reverse Transcription Polymerase Chain Reaction (qRT-PCR) was utilized in miRNA profiling of circulatory exosomes. Evaluation of study quality using the NOS scale demonstrated the high quality of all articles with scores of either 7 or 8 (Figure S1). However, evaluation of the enrolled studies according to the MISEV guidelines revealed a score range from 3 to 8 (Table 1).

### 3.2. Diagnostic Value of Exosome-Derived miRNAs

**Figure 2:**
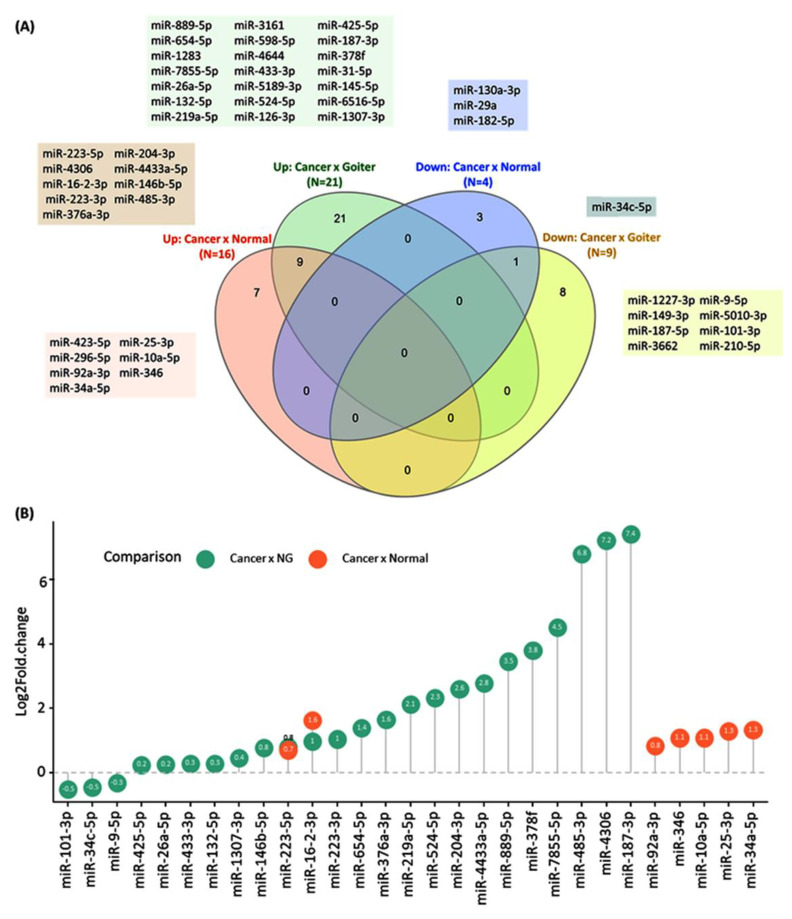
Deregulated exosomal miRNAs in thyroid cancer. (**A**) Venn diagram shows the intersection between aberrantly expressed miRNAs across studies with different comparisons [25,26,28,30,31,32,33,34,35]. The Venn diagram was plotted online (http://www.interactivenn.net/) (accessed on 15 July 2021) [36]. (**B**) The lollipop plot shows the fold change of deregulated miRNAs in datasets comparing cancer versus normal and cancer versus nodular goiter. Only miRNAs with reported expression level values are shown [30,32,33,35]. R package ‘ggplot2′ and ‘ggpubr’ were used.

A total of 49 exosomal miRNAs (37 up and 12 down) were significantly deregulated in the circulation of thyroid cancer patients. Pooled meta-analysis of studies showed 16 upregulated miRNAs [30,32,33,34,35] and four downregulated miRNAs [26,28,32] in the circulatory exosomes of cancer versus normal subjects. To compare cancer and nodular goiter, 21 upregulated [25,31,32,33] and 9 downregulated miRNAs [31,32] were observed. The intersection between both types of comparisons yielded one downregulated (miR-34c-5p) and nine upregulated miRNAs (miR-223-5p, miR-4306, miR-16-2-3p, miR-223-3p, miR-376a-3p, miR-204-3p, miR-4433a-5p, miR-146b-5p, and miR-485-3p) consistently differentially expressed (Figure 2A). Of those with reported expression levels, miR-187-3p, miR-4306, and miR-485-3p had the highest values, while miR-101-3p, miR-34c-5p, and miR-9-5p were under-expressed in cancer compared to goiter (Figure 2B). Abstracted raw data are demonstrated in Table S2.

**Figure 3:**
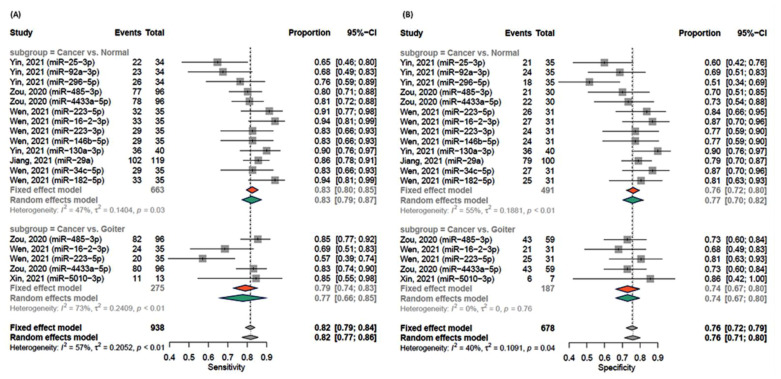
Sensitivity and specificity of exosomal miRNAs stratified by type of comparison. Each row in the forest plot represented the miRNA result of a study/dataset. Estimate and confidence intervals are shown as box and bar. Pooled result of the subgroup analysis is shown separately in red (cancer versus normal) and green (cancer versus nodular goiter) diamonds. Heterogeneity was assessed using the Q test, and the magnitude of heterogeneity was quantified using I2. If I2 exceeded 50%, heterogeneity across studies was reported, and random-effects model results were considered; otherwise, a fixed-effects model was used. The final overall results are illustrated in the lower panel with grey diamonds. (**A**) Forest plot for the sensitivity of testing exosomal miRNAs. (**B**) Forest plot for the specificity of testing exosomal miRNAs. R package ‘meta’ was used.

Diagnostic accuracy measures were able to be estimated for eighteen miRNAs of the reported studies. All miRNAs were analyzed in the Asian population and were quantified using qRT-PCR assays. Figure 3 summarizes the sensitivity and specificity of each miRNA, pooled by comparison type and summarized as overall estimates. Pooled sensitivity was 82% (95%CI = 77–86%). It was higher comparing cancer to normal (83%, 95%CI = 79–87%) than cancer to nodular goiter 77% (95%CI = 66–85%) (Figure 3A). Overall pooled specificity was 76% (95%CI = 71–80%). Consistently, higher specificity was observed when compared to normal subjects (77%, 95%CI = 70–82%) than goiter patients (74%, 95%CI = 67–80%) (Figure 3B). The best biomarkers with high sensitivity were miR-16-2-3p (94%), miR-223-5p (91%), miR-130a-3p (90%), and miR182-5p (94%). Similarly, they showed high specificity (87%, 84%, 90%, and 81%, respectively) in addition to miR-34c-5p (87%).

There was homogeneity across studies for specificity analysis; however, mild heterogeneity was detected in the sensitivity analysis due to lower diagnostic performance than cancer and goiter cohorts. The source of heterogeneity was traced by analysis of the diagnostic threshold. Visual inspection of forest plots showed that the inverse relationship between pairs of accuracy estimates (sensitivity and specificity, positive and negative likelihood ratios) was absent. There was no significant correlation between true positive and false positive rates (Spearman’s correlation coefficient = −0.273, *p* = 0.27), indicating the absence of threshold effect. Meta-regression analysis revealed the insignificant influence of the type of comparison on detected heterogeneity (coefficient = −0.41, *p* = 0.48).

**Figure 4:**
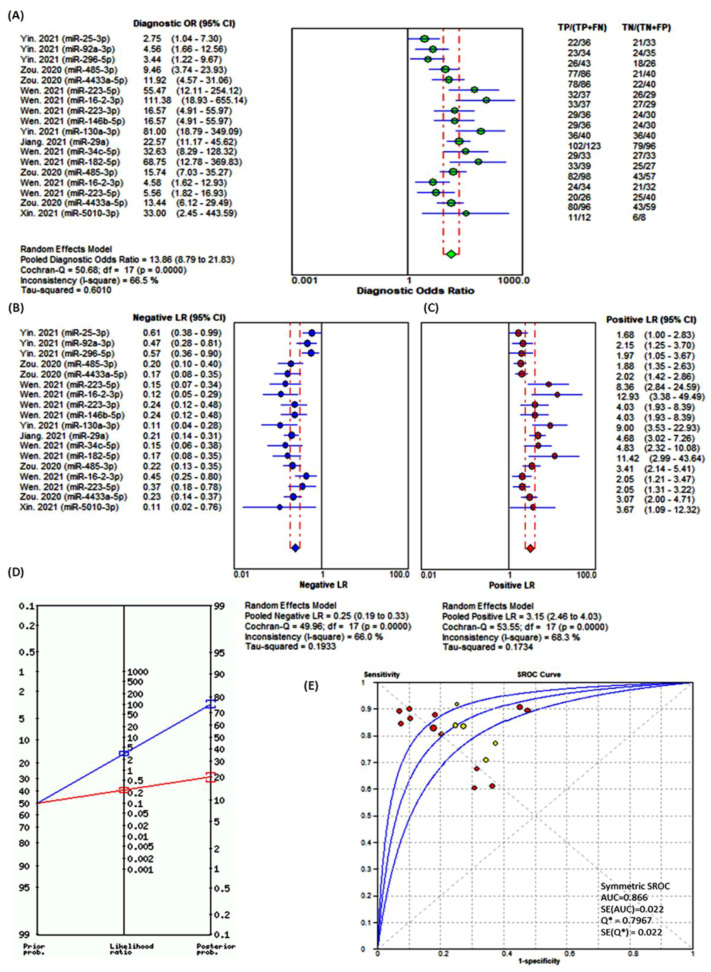
Effectiveness of exosomal miRNAs as a diagnostic test. All tested miRNAs were upregulated in cancer patients except miR-130a-3p, miR-29a, miR-34c-5p, miR-182, 5p, and miR-5010-3p. (**A**) Diagnostic odds ratio. It is defined as the odds of the test being positive if the subject has cancer relative to the odds of the test being positive if the subject does not have the disease (=PLR/NLR). The higher diagnostic odds ratios are indicative of better test performance. The DerSimonian–Laird pooling method was used [37]. (**B**) Negative likelihood ratio. It gives the odds of having a diagnosis in patients with a negative test. The change is in the form of a ratio, usually less than 1. The smaller the -LR, the more informative the test. (**C**) Positive likelihood ratio. It is the ratio of the probability that a positive test result is expected in a diseased individual to the probability that a positive result occurs in a healthy subject. It tells us how many times it is more likely to observe a positive test result in a diseased than in a healthy individual. The more the likelihood ratio for a positive test (+LR) is greater than 1, the more likely the disease is. (**D**) Fagan’s Bayesian nomogram for the 18 combined miRNA panel. Lines are then drawn from the prior probability on the left through the likelihood ratios in the center and extended to the posterior probabilities on the right. Pretest probability on the left vertical line, likelihood ratio in the middle vertical line. The predicted posttest probability is on the right vertical line. Pooled results showed a moderate shift in posttest probability. With the prior probability of 50%, the probability of the disease increases to 76% (95%CI = 74–78%) with positive test results and decreases the probability of having the disease to 20% (95%CI = 17–22%) in the presence of a negative test. (**E**) Summary ROC curve. It is created by plotting the true positive rate (sensitivity) against the false positive rate (1-specificity). Symmetric sROC curve fitted using Moses’ Model (weighted regression: inverse variance). Significant miRNA testing for cancer versus normal in red circles and for cancer versus nodular goiter in yellow circles. The position of the dots depends on their discriminatory ability; the more accurate the test is, the closer the curve to the upper left-hand corner of the ROC plot. The middle blue line indicates the estimated sROC curve, surrounded by two other lines for the 95% confidence region for the summary estimate. Q* is the point of the curve in which sensitivity equals specificity. Meta-DiSc v1.4 was used for meta-analysis (https://meta-disc.software.informer.com/1.4/) (accessed on 8 June 2021) [38].

The trade-off between sensitivity and specificity was examined by estimating the diagnostic odds ratio to compare the performance of miRNA tests. Overall analysis showed a DOR of 13.6 (95%CI = 8.8–21.8). The best four markers were miR-16-2-3p (DOR = 11.4, 95%CI = 18.9–655.1), miR-130a-3p (DOR = 81.0, 95%CI = 18.9–349.1), miR-182-5p (DOR = 68.8, 95%CI = 12.8–369.8), and miR-223-5p (DOR = 55.5, 95%CI = 12.1–254.1) (Figure 4A). The pooled negative likelihood ratio of reported miRNAs was 0.25 (95%CI = 0.19–0.33), indicating a 4-fold decrease in the odds of having cancer in a patient with a negative test result. Significant heterogeneity between miRNAs was observed (I2 = 66.0%, *p* < 0.001). Across studies, the best markers for excluding cancer from normal individuals in the presence of negative test results were miR-130a-3p (NLR = 0.11, 95%CI = 0.04–0.28) followed by miR-16-2-3p (NLR = 0.12, 95%CI = 0.05–0.29), and for discriminating cancer from nodular goiter patients was miR-5010-3p (NLR = 0.11, 95%CI = 0.02–0.76) (Figure 4B). The overall positive likelihood ratio was 3.15 (95%CI = 2.46–4.03), which would indicate a 3-fold increase in the odds of having cancer in a patient with a positive test. The best significant increase in the probability of cancer was found in the case test results that yielded overexpression of miR-16-2-3p (PLR = 12.9, 95%CI = 3.4–49.5) and miR-223-5p (PLR = 8.36, 95%CI = 2.8–24.6) and lower levels of miR-182-5p (PLR = 11.4, 95%CI = 2.9–43.6). There was remarkable heterogeneity across testing of the 18 exosomal miRNAs reported (I2 = 68.3%, *p* < 0.001) (Figure 4C). Based on the pooled negative and positive likelihood ratios and prior probability value of 50%, the probability of the disease increases to 76% (95%CI = 74–78%) with positive test results and decreases the probability of having the disease to 20% (95%CI = 17–22%) in the presence of a negative test. Fagan’s Bayesian nomogram is shown in Figure 4D. The hierarchical summary ROC (sROC) model jointly summarizes sensitivity and specificity irrespective of the threshold effect of different miRNA tests. The area under the sROC curve was 0.866 ± 0.022 (Figure 4E).

**Figure 5:**
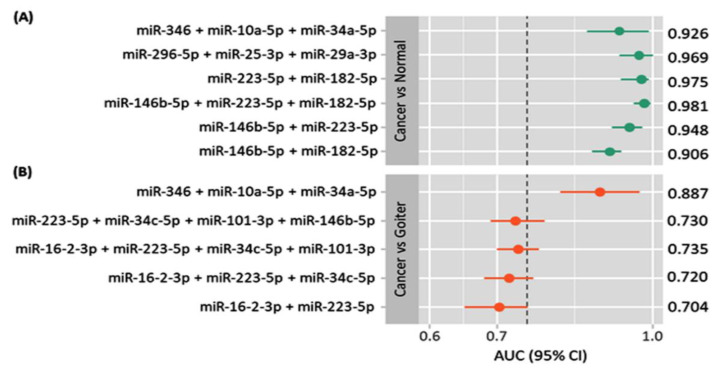
Comparison of the diagnostic accuracy of various miRNA-based panels. Area under the curve (AUC) and 95% confidence interval (CI) of the receiver operator characteristic curve analysis for each panel are plotted [30,32,35]. The threshold for optimum diagnostic accuracy was set at 0.75. Subgroup analysis for exosomal miRNA expression was carried out based on the type of comparison. (**A**) Cancer compared to normal subjects; (**B**) cancer compared to nodular goiter.

As depicted in Figure 5, various miRNA panels were suggested in the literature. Six panels of two to four exosomal miRNAs showed higher diagnostic value with AUC ranging from 0.906 to 0.981. The best discriminative ability to differentiate between cancer and non-cancer individuals was observed for miR-146b-5p + miR-223-5p + miR-182-5p (AUC = 0.981, sensitivity = 93.8% (84.9–98.3), specificity = 92.9% (76.5–99.1) at cutoff > 0.769), followed by miR-223-5p + miR-182-5p (AUC = 0.975, sensitivity = 90.8% (80.9–96.5), specificity = 96.4% (81.6–99.9) at cutoff > 0.855) [32]. The De Long test showed no significant difference between different combinations (*p* > 0.05). Whereas, the triple biomarker, miR-346 + miR-10a-5p + miR-34a-5p, demonstrated high performance to discriminate between cancer and benign nodular goiter disease (AUC = 0.887, 95%CI = 0.81–0.97) [35] (Table S3).

### 3.3. Prognostic Value of Exosome-Derived miRNAs

Across studies [21,32,33], some miRNAs were reported to be associated with poor prognostic outcomes (Table 2). Thirteen miRNAs were significantly downregulated in patients with lymph node metastasis, including miR-130a-3pmiR-1915, miR-323a-5p, miR-543, and miR-381-3p. Elevated levels of 12 miRNAs were associated with lymph node metastasis, including miR-485-3p, miR-221-3p, miR-222-3p, miR-146b-5p, and miR-21-5p (Table S4).

**Figure 6:**
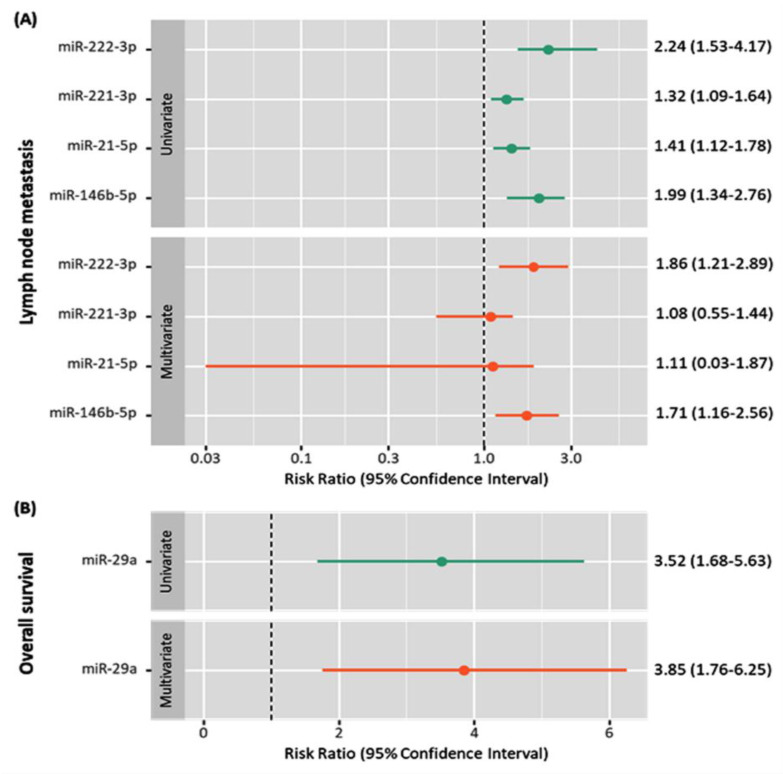
Univariate and multivariate analyses of the prognostic factors for exosomal miRNAs. (**A**) for the lymph node metastasis; (**B**) for the overall survival. Data are shown as relative risk and 95% confidence interval for univariate and multivariate analyses [21,28].

Considering studies reporting multivariate analysis for predicting survival and lymph node biopsy [21,28], the relative risk and 95% confidence interval for univariate and multivariate analyses are reported in Figure 6. Lower expression of miR-29a was associated with shorter survival times and nearly 4-fold increased risk of mortality (RR = 3.85, 95%CI = 1.76–6.25, *p* = 0.010), whereas the upregulated profiles of miR-146b-5p (RR = 1.71, 95%CI = 1.16–2.56, *p* = 0.012) and miR-222-3p (RR = 1.86, 95%CI = 1.21–2.89, *p* = 0.002) were independent predictors for lymph node metastasis in thyroid cancer.

As depicted in Table 3, the overexpression of miR-204-3p had a higher ability to differentiate patients with large tumor size > 1 cm (AUC = 0.798, 95%CI = 0.71–0.88). Upregulated miR-485-3p was associated with extrathyroidal extension (AUC = 0.726, 95%CI = 0.62–0.83), BRAF mutation (AUC = 0.890, 95%CI = 0.83–0.96), and advanced clinical stage (AUC = 0.753, 95%CI = 0.65–0.86) [33]. Another miRNA, miR-29a had similar discrimination ability to predict the late stage (AUC = 0.758, 95%CI = 0.70–0.81), which was not significant from miR-485-3p (De Long test: *p* > 0.05). Lower expression of exosomal miR-29a was also associated with recurrence prediction (AUC = 0.753, 95%CI = 0.68–0.80) [28].

Due to the limited available data and the small number of studies, we could not perform subgroup analysis categorized by population demographics, histological subtypes, disease stage, mutation status, initial curative therapy, or response to treatment.

### 3.4. Functional Enrichment Analysis

**Figure 7:**
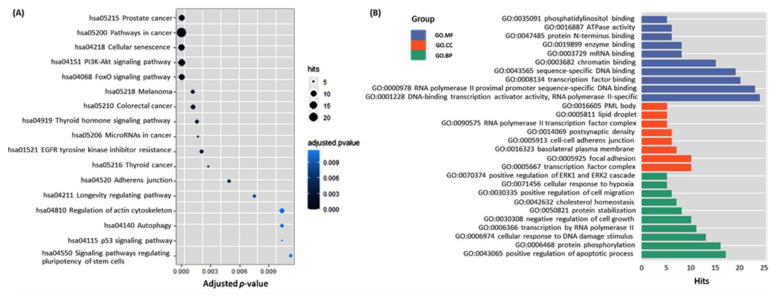
Functional enrichment analysis. (**A**) KEGG pathway enrichment. Top enriched pathways are represented in a scatter plot. The vertical axis represents the pathname, and the horizontal axis represents the q-value. The color of the point represents the size of the q-value. The number of differential genes included in each pathway is expressed by the size of the point. (**B**) The vertical axis represents the gene ontology term, and the horizontal axis represents the number of gene targets (hits) for 3′UTR, CDS, and 5′UTR in that GO. The top significantly enriched terms with q values < 0.05 were considered. Data source: MirWalk 3.0 (http://mirwalk.umm.uni-heidelberg.de/) (accessed on 12 June 2021), using the following filter: 0.95, Targetscan, miRDB, and miRTarbase.

Around 50 miRNAs were included in the current meta-analysis. To unleash their potential mechanism in cancer, we performed KEGG and gene ontology analyses with the experimentally validated target genes of miRNAs. KEGG analysis demonstrated that the miRNAs might play an important part in pathways closely related to thyroid cancer (e.g., thyroid cancer pathway, pathways in cancer, miRNAs in cancer, p53 signaling pathway, etc. GO analysis showed that the miRNAs could also regulate important biological processes, including DNA damage response, cell growth, apoptosis, and response to hypoxia. Results of KEGG and GO analyses for miRNAs are presented in Figure 7. Target genes in each KEGG pathway are demonstrated in Table S5.

## 4. Discussion

Recently, research on exosomes and TC prognosis has become a medical hotspot. Many studies have found that exosomes play a vital role in the diagnosis/prognosis and treatment of TC, although the results remain controversial [25,27,31]. To our knowledge, this is the first systematic review and meta-analysis evaluating the diagnostic and prognostic value of exosomal miRNAs in thyroid cancer. Our meta-analysis consists of 12 articles, including 1164 patients and 540 controls. The pooled sensitivity was 82% (95%CI = 77–86%), pooled specificity was 76% (95%CI = 71–80%), and pooled DOR was 13.6 (95%CI = 8.8–21.8). The best biomarkers with high sensitivity were miR-16-2-3p (94%), miR-223-5p (91%), miR-130a-3p (90%), and miR182-5p (94%). Similarly, they showed high specificity, in addition to miR-34c-5p. This indicates that miRNAs can be potentially useful biomarkers when used as a diagnostic tool for thyroid cancer.

MiRNAs play a key role in various processes, including cancer development, progression of the disease, and metastasis [39]. These highly conserved molecules are exceptionally stable in blood and urine due to their small size and resistance to nucleolytic cleavage by RNAse [40]. This feature allows miRNAs to be a reliable, non-invasive, and sensitive method of detecting tumors. Furthermore, miRNA exhibit unique “molecular signatures.” These mutations can be used to identify a wide range of malignancies, including hepatocellular, lung, and thyroid cancer [41,42,43]

More studies are emerging on circulating miRNA for detecting TC [18,21,32,44]. For instance, Liu et al. conducted a meta-analysis and found that circulating miR-222 and miR-146b had high diagnostic value for PTC in the Asian population [18]. Specifically, miR-222 had a sensitivity of 0.70%, specificity of 0.90%, and a diagnostic ratio of 22.55. Other miRNAs reported to be associated with thyroid cancer include miR-146b and miR-221, which are upregulated in benign and malignant thyroid nodules [45,46,47]. MiR-146b can serve as an independent risk factor for poor prognosis in PTCs. However, overexpression of miR-146b can be found in both PTCs and FTCs and cannot help differentiating between tumors [45]. Additionally, Samsonov et al. confirmed that plasma exosomal miR-21 could help differentiate benign tumors and FTC [25]. Our results add miR-21, miR-451a, miR-1290, and miR-638 to the existing repertoire of miRNAs that can be used as diagnostic tools for TC.

Our results support previous studies demonstrating that a panel of multiple miRNA assays has higher diagnostic accuracy than single miRNA assays [18,48,49]. The best discriminative ability to differentiate between cancer and non-cancer individuals was an miR-146b-5p + miR-223-5p + miR-182-5p panel. Thus, it is important to consider using a combination of miRNA rather than single miRNAs when using these biomarkers as a diagnostic tool.

The utility of miRNAs is extensive as they can serve as prognostic markers for TNM staging, tumor size, short-term survival, overall survival, and recurrence [27,50]. Our study adds to the existing literature by demonstrating that circulating exosomal miR-21, miR-451a, miR-1290, and miR-638 can be used to predict OS and DFS in these patients further. Jiang et al. described exosomal miR-146-5p and miR-222-3p to be upregulated in PTC with LNM [21]. Overexpression of these various miRNA may play a role in the migration and invasion of PTC. By further deciphering the roles of miRNAs in cancer outcomes, such as lymph node metastasis, surgical interventions can be limited. For instance, prophylactic neck dissection is controversial in patients with clinically LNM-negative PTC patients. Thus, non-invasive biomarkers can help prevent unnecessary surgery while providing information on prognosis [51].

The biomarkers with highest sensitivity in our study were miR-16-2-3p (94%), miR-223-5p (91%), miR-130a-3p (90%), and miR182-5p. Liang et al. similarly reported that miR-16-2-3p and miR-223-5p could be utilized for detecting PTC from benign nodules [32]. MiR130a-3p has been previously studied in glioblastoma, which regulates disease progression [52]. We found that miR-182-5p underexpression was associated with TC. MiR-182-5p has been studied in other cancers, including hepatocellular cancer and breast cancer, where it is proposed to be responsible for the proliferation and metastasis of cancer [53,54]. In thyroid cancer, other studies have shown that miR-182-5p can be a helpful marker for PTC, particularly metastasis, which agrees with our results [55].

The clinical advantages of miRNAs are multi-fold. First, miRNAs can be used as a screening tool for early detection of PTC, which would aid in early cancer prevention and improve patient survival. Secondly, the use of these biomarkers can help prevent unnecessary diagnostic surgery. Using the Bethesda classification of thyroid nodule fine-needle aspiration, 20–30% of thyroid nodules are considered “indeterminate” (Bethesda Class III/IV), and approximately 15–30% of these that are surgically removed are malignant [56]. Therefore, most patients who undergo surgery under these classifications have benign diseases and do not require surgery. By using miRNA, we have the potential to save patients from the burden of surgery.

This study had some limitations. First, except for one study, all studies included in our analysis originated from China and included an Asian population. We recommend that future studies include other ethnicities to improve the generalization of the miRNAs panel. Secondly, studies involving biomarkers should expand their analysis to demonstrate comprehensive diagnostic accuracy measures. Thirdly, measures of test accuracy are not fixed properties of a test, and there are generally many contributing factors leading to variation. Variation between studies in following the MISEV guidelines [35] on the experimental methodology can add a further element of heterogeneity that also should be considered. Therefore, heterogeneity is a common feature of DTA reviews. Due to this wide variability or heterogeneity between studies, we suggest that future studies narrow their analysis to select the best miRNAs and follow the standard published methodology for reporting their results.

Future research should continue to evaluate the causative role of these miRNAs in thyroid cancer development. By understanding the underlying mechanisms in which miRNAs affect tumor progression or metastasis, we can better develop therapeutics using miRNAs. For instance, studies have shown that exosomal miRNA-423-5p secreted by PTC can, in turn, deliver the miRNA into PTC cells [34]. Thus, there is potential to use exosomal miRNAs as therapeutic targets in PTC. Furthermore, we recommend that a panel of exosomal miRNAs is tested and validated.

## 5. Conclusions

We are the first to report on circulating exosomal miRNA as a promising diagnostic tool to distinguish between malignant and benign thyroid cancer. Specifically, miR-16-2-3p, miR-223-5p, miR-130a-3p, and miR182-5p can provide high diagnostic value with high sensitivity and specificity. We recommend that circulating exosomal miRNAs be considered potential biomarkers to establish diagnostic and prognostic information for thyroid cancer.

## Figures and Tables

**Table 1:** The main features of eligible studies in the systemic review.

First Author	Year	Country	No of Patients	No of Controls	Test Method	Name of PCR Kit	Method of Exosomes Isolation	MISEV	Target Exosome miRNA	Tumor Subtype	Mean Age, Y	Female (%)	Ref.
Yin	2021	China	40	40	qRT-PCR (SYBR Green)	SYBR Green Super mix (Bio-Rad Laboratories, Inc.)	Invitrogen™ Exosome Isolation Kit (Thermo Fisher Scientific, Inc.)	7	miR-130a-3p	DTC	64.9	27.5%	[26]
Xin	2021	China	491		--	Not estimated	--	--	miR-129-2 miR-889	PTC	--	--	[27]
Wen	2021	China	119	100	qRT-PCR	TaqMan MicroRNA RT Kit (Applied Biosystems)	ExoQuick Exosome Precipitation Solution (System Biosciences)	3	miR-29a	PTC	--	52.1%	[28]
Li	2021	China	--		qRT-PCR	Not estimated	--	--	miR-148a-3p	DTC	--	--	[29]
Zou	2020	China	100	96	qRT-PCR	SYBR Green (SYBR^®^ Premix Ex TaqTM II, TaKaRa, Dalian, China).	ExoQuick Exosome Precipitation Solution (System Biosciences, Mountain View, CA, USA).	6	miR-25-3p miR-296-5p miR-92a-3p	PTC	--	--	[30]
Pan	2020	China	13	7	Small RNA sequencing	TruSeq SR Cluster Kit v3-cBot-HS (Illumina, San Diego, CA, USA)	Exosomes were isolated from the plasma through ultracentrifugation method.	7	miR-5189-3p miR-5010-3p miR-598-5p miR-3161 miR-6516-5p miR-4644 miR-1283 miR-1227-3p miR-149-3p miR-210-5p miR-3662 miR-187-5p	PTC	--	100%	[31]
Liang	2020	China	51	69	qRT-PCR	SYBR Green PCR Kit (QIAGEN)	Exosome Precipitation Solution (EXOQ20A-1, SBI, Mountain View, CA, USA)	8	miR-16-2-3p miR-223-5p miR-34c-5p miR-182-5p miR-223-3p miR-146b-5p miR-16-2-3p miR-223-5p	PTC	44.0	51.4%	[32]
Jiang	2020	China	64		qRT-PCR	Not determined	Not determined	--	miR-146b-5p miR-221-3p miR-222-3p miR-21-5p miR-204-5p	PTC	41.2	78.1%	[21]
Dai	2020	China	96	30	qRT-PCR	MiR-X miRNA qRTPCR SYBR Kit (Takara) and miDETECT A Track™ miRNA RT-qPCR Primers (Ribobio).	Exosomes were isolated with a combination of centrifugation and ultracentrifugation.	5	miR-485-3p miR-4433a-5p miR-4306 miR-376a-3p miR-204-3p	PTC	56.6	--	[33]
Ye	2019	China	60	30	qRT-PCR	miScript SYBR Green PCR Kit (Qiagen, Germany).	Exosomes were isolated with ultracentrifugation.	5	miRNA423-5p	PTC	--	--	[34]
Wang	2019	China	120	160	qRT-PCR	The expression levels of miRNAs in plasma and exosomes were measured using SYBR Green dye	Exosomes of peripheral plasma were isolated by using ExoQuick™ (System Biosciences, Mountain View, CL, USA)	6	miR-346 miR-10a-5p miR-34a-5p	PTC	--	--	[35]
Samsonov	2016	Russia	10	8	qRT-PCR	qPCR was performed using Cancer Focus microRNA PCR Panels and ExiLENT SYBR Green master mix (both from Exiqon, Denmark) on CFX96 Touch™ Real-Time PCR Detection System (Bio-Rad, USA).	Exosomes were isolated with centrifugation method.	5	miR-21 miR-181a	PTC	54.5	80%	[25]

DTC: well-differentiated thyroid cancer; MISEV: minimal information for studies of extracellular vesicles [24]; PTC: papillary thyroid cancer; qRT-PCR: quantitative Real-Time Polymerase Chain Reaction.

**Table 2:** Association of exosomal miRNAs with poor prognostic features in thyroid cancer patients.

miRNA	Expression	LNM	TNM Stage	Tumor Size	ETE	BRAF Mutation	Short Survival	Recurrence	Ref.
miR-130a-3p	Low	(+)	(+)	(+)					[26]
miR-29a	Low		(+)	(+)	(+)		(+)	(+)	[28]
miR-148a-3p	Low	(+)		(+)					[29]
miR-146b-5p	High	(+)					(+)		[21]
miR-222-3p	High	(+)					(+)		[21]
miR-423-5p	High	(+)							[34]
miR-204-3p	High			(+)					[33]
miR-4306	Low			(+)					[33]
miR-4433a-5p	High	(+)	(+)		(+)	(+)			[33]
miR-485-3p	High	(+)	(+)	(+)	(+)	(+)			[33]
miR-21-5p	High	(+)							[21]
miR-204-5p	High	(+)							[21]
miR-221-3p	High	(+)							[21]
miR-182-5p	High	(+)							[32]
miR-26b-5p	High	(+)							[32]
miR-126-3p	High	(+)							[32]
miR-542-3p	High	(+)							[32]
miR-32-5p	High	(+)							[32]
miR-363-3p	High	(+)							[32]
miR-1912	Low	(+)							[32]
miR-323a-5p	Low	(+)							[32]
miR-543	Low	(+)							[32]
miR-381-3p	Low	(+)							[32]
miR-128-3p	Low	(+)							[32]
miR-139-5p	Low	(+)							[32]
miR-885-3p	Low	(+)							[32]
miR-409-5p	Low	(+)							[32]
miR-28-5p	Low	(+)							[32]
miR-151a-5p	Low	(+)							[32]
miR-490-3p	Low	(+)							[32]

LNM: lymph node metastasis; ETE: extrathyroidal extension, BRAF mutation: missense mutation V600E. (+): positive association.

**Table 3:** Receiver operator characteristic curve analysis for assessment of the prognostic performance of exosomal microRNAs.

	miRNAs	Cases	Controls	Expression	AUC	Lower	Upper	Ref.
Tumor size ≥ 1 cm vs. <1 cm	miR-204-3p	56	40	High	0.798	0.71	0.88	[33]
ETE vs. none	miR-485-3p	59	37	High	0.726	0.62	0.83	[33]
BRAF mutation vs. wild type	miR-485-3p	65	31	High	0.890	0.83	0.96	[33]
Late stage vs. stage I/II	miR-485-3p	33	63	High	0.753	0.65	0.86	[33]
	miR-29a	41	78	Low	0.758	0.70	0.81	[28]
Recurrence vs. none	miR-29a	30	89	Low	0.753	0.68	0.80	[28]

The area under the curve (AUC) and 95% confidence interval (lower and upper limits) are reported.

## Data Availability

The data presented in this study are available in the manuscript and Supplementary Materials.

## Supplementary Materials

The following are available online at https://www.mdpi.com/article/10.3390/cancers13174295/s1, Figure S1: Quality assessment for included studies. Evaluation using the Newcastle–Ottawa scale was carried out. Table S1: Major recommendations of MISEV, Table S2: Deregulated exosomal miRNAs in thyroid cancer, Table S3: Diagnostic role of combined miRNA panels in thyroid cancer, Table S4: Exosomal miRNAs associated with lymph node metastasis in thyroid cancer patients. Table S5. List of target genes of KEGG pathways enriched in thyroid cancer exosomal miRNA meta-signature.

## References

[1] Siegel R.L., Miller K.D., Jemal A. (2018). Cancer statistics, 2018. CA Cancer J. Clin..

[2] Siegel R.L., Miller K.D., Jemal A. (2017). Cancer statistics, 2017. CA Cancer J. Clin..

[3] Yoon R.G., Baek J.H., Lee J.H., Choi Y.J., Hong M.J., Song D.E., Kim J.K., Yoon J.H., Kim W.B. (2014). Diagnosis of Thyroid Follicular Neoplasm: Fine-Needle Aspiration Versus Core-Needle Biopsy. Thyroid.

[4] Guille J.T., Opoku-Boateng A., Thibeault S.L., Chen H. (2014). Evaluation and Management of the Pediatric Thyroid Nodule. Oncologist.

[5] Papini E., Guglielmi R., Bianchini A., Crescenzi A., Taccogna S., Nardi F., Panunzi C., Rinaldi R., Toscano V., Pacella C.M. (2002). Risk of Malignancy in Nonpalpable Thyroid Nodules: Predictive Value of Ultrasound and Color-Doppler Features. J. Clin. Endocrinol. Metab..

[6] Caraway N.P., Sneige N., Samaan N.A. (1993). Diagnostic pitfalls in thyroid fine-needle aspiration: A review of 394 cases. Diagn. Cytopathol..

[7] Mongelli M.N., Giri S., Peipert B.J., Helenowski I.B., Yount S.E., Sturgeon C. (2020). Financial burden and quality of life among thyroid cancer survivors. Surgery.

[8] Rezig L., Servadio A., Torregrossa L., Miccoli P., Basolo F., Shintu L., Caldarelli S. (2018). Diagnosis of post-surgical fine-needle aspiration biopsies of thyroid lesions with indeterminate cytology using HRMAS NMR-based metabolomics. Metabolomics.

[9] Toraih E.A., Ibrahiem A.T., Fawzy M.S., Hussein M.H., Al-Qahtani S.A.M., Shaalan A.A.M. (2017). MicroRNA-34a: A Key Regulator in the Hallmarks of Renal Cell Carcinoma. Oxid. Med. Cell. Longev..

[10] Toraih E.A., Aly N.M., Abdallah H.Y., Al-Qahtani S.A., Shaalan A.A., Hussein M.H., Fawzy M.S. (2017). MicroRNA—Target cross-talks: Key players in glioblastoma multiforme. Tumor Biol..

[11] Heneghan H., Miller N., Kerin M. (2010). MiRNAs as biomarkers and therapeutic targets in cancer. Curr. Opin. Pharmacol..

[12] Cuellar T.L., McManus M.T. (2005). MicroRNAs and endocrine biology. J. Endocrinol..

[13] Lima C.R., Geraldo M.V., Fuziwara C.S., Kimura E.T., Santos M.F. (2016). MiRNA-146b-5p upregulates migration and invasion of different Papillary Thyroid Carcinoma cells. BMC Cancer.

[14] Swierniak M., Wojcicka A., Czetwertynska M., Stachlewska E., Maciag M., Wiechno W., Gornicka B., Bogdanska M., Koperski L., De La Chapelle A. (2013). In-Depth Characterization of the MicroRNA Transcriptome in Normal Thyroid and Papillary Thyroid Carcinoma. J. Clin. Endocrinol. Metab..

[15] Colombo M., Raposo G., Théry C. (2014). Biogenesis, secretion, and intercellular interactions of exosomes and other extracellular vesicles. Annu. Rev. Cell Dev. Biol..

[16] Schwarzenbach H., Nishida N., Calin G., Pantel K. (2014). Clinical relevance of circulating cell-free microRNAs in cancer. Nat. Rev. Clin. Oncol..

[17] Cheng L., Sun X., Scicluna B.J., Coleman B.M., Hill A. (2014). Characterization and deep sequencing analysis of exosomal and non-exosomal miRNA in human urine. Kidney Int..

[18] Liu Y., Geng H., Liu X., Cao M., Zhang X. (2021). A meta-analysis of circulating microRNAs in the diagnosis of papillary thyroid carcinoma. PLoS ONE.

[19] Shao N., Xue L., Wang R., Luo K., Zhi F., Lan Q. (2018). miR-454-3p Is an Exosomal Biomarker and Functions as a Tumor Suppressor in Glioma. Mol. Cancer Ther..

[20] Hannafon B.N., Trigoso Y.D., Calloway C.L., Zhao Y.D., Lum D.H., Welm A.L., Zhao Z.J., Blick K.E., Dooley W.C., Ding W.Q. (2016). Plasma exosome microRNAs are indicative of breast cancer. Breast Cancer Res..

[21] Jiang K., Li G., Chen W., Song L., Wei T., Li Z., Gong R., Lei J., Shi H., Zhu J. (2020). Plasma Exosomal miR-146b-5p and miR-222-3p are Potential Biomarkers for Lymph Node Metastasis in Papillary Thyroid Carcinomas. OncoTargets Ther..

[22] Page M.J., McKenzie J.E., Bossuyt P.M., Boutron I., Hoffmann T.C., Mulrow C.D., Shamseer L., Tetzlaff J.M., Akl E.A., Brennan S.E. (2021). The PRISMA 2020 statement: An updated guideline for reporting systematic reviews. BMJ.

[23] Elshazli R.M., Toraih E.A., Elgaml A., El-Mowafy M., El-Mesery M., Amin M., Hussein M.H., Killackey M., Fawzy M.S., Kandil E. (2020). Diagnostic and prognostic value of hematological and immunological markers in COVID-19 infection: A meta-analysis of 6320 patients. PLoS ONE.

[24] Witwer K.W., Soekmadji C., Hill A.F., Wauben M.H., Buzás E.I., Di Vizio D., Falcon-Perez J.M., Gardiner C., Hochberg F., Kurochkin I.V. (2017). Updating the MISEV minimal requirements for extracellular vesicle studies: Building bridges to reproducibility. J. Extracell. Vesicles.

[25] Samsonov R., Burdakov V., Shtam T., Radzhabova Z., Vasilyev D., Tsyrlina E., Titov S., Иванов М., Berstein L., Filatov M. (2016). Plasma exosomal miR-21 and miR-181a differentiates follicular from papillary thyroid cancer. Tumor Biol..

[26] Yin G., Kong W., Zheng S., Shan Y., Zhang J., Ying R., Wu H. (2021). Exosomal miR-130a-3p promotes the progression of differentiated thyroid cancer by targeting insulin-like growth factor 1. Oncol. Lett..

[27] Xin Y., Meng K., Guo H., Chen B., Zheng C., Yu K. (2021). Exosomal hsa-miR-129-2 and hsa-miR-889 from a 6-microRNA signature might be a potential biomarker for predicting prognosis of papillary thyroid carcinoma. Comb. Chem. High Throughput Screen..

[28] Wen Q., Wang Y., Li X., Jin X., Wang G. (2021). Decreased serum exosomal miR-29a expression and its clinical significance in papillary thyroid carcinoma. J. Clin. Lab. Anal..

[29] Li S., Zhang S., Yang W., Li F., Long H. (2021). Diagnostic Value of Exosomal miR-148a-3p in the Serum of Patients with Differentiated Thyroid Cancer. Clin. Lab..

[30] Zou X., Gao F., Wang Z.-Y., Zhang H., Liu Q.-X., Jiang L., Zhou X., Zhu W. (2020). A three-microRNA panel in serum as novel biomarker for papillary thyroid carcinoma diagnosis. Chin. Med. J..

[31] Pan Q., Zhao J., Li M., Liu X., Xu Y., Li W., Wu S., Su Z. (2019). Exosomal miRNAs are potential diagnostic biomarkers between malignant and benign thyroid nodules based on next-generation sequencing. Carcinogenesis.

[32] Liang M., Yu S., Tang S., Bai L., Cheng J., Gu Y., Li S., Zheng X., Duan L., Wang L. (2020). A Panel of Plasma Exosomal miRNAs as Potential Biomarkers for Differential Diagnosis of Thyroid Nodules. Front. Genet..

[33] Dai D., Tan Y., Guo L., Tang A., Zhao Y. (2020). Identification of exosomal miRNA biomarkers for diagnosis of papillary thyroid cancer by small RNA sequencing. Eur. J. Endocrinol..

[34] Ye W., Deng X., Fan Y. (2019). Exosomal miRNA423-5p mediated oncogene activity in papillary thyroid carcinoma: A potential diagnostic and biological target for cancer therapy. Neoplasma.

[35] Wang Z., Lv J., Zou X., Huang Z., Zhang H., Liu Q., Jiang L., Zhou X., Zhu W. (2019). A three plasma microRNA signature for papillary thyroid carcinoma diagnosis in Chinese patients. Gene.

[36] Heberle H., Meirelles G.V., Da Silva F.R., Telles G.P., Minghim R. (2015). InteractiVenn: A web-based tool for the analysis of sets through Venn diagrams. BMC Bioinform..

[37] Russell P.S.S., Chikkala S.M., Earnest R., Viswanathan S.A., Russell S., Mammen P.M. (2020). Diagnostic accuracy and clinical utility of non-English versions of Edinburgh Post-Natal Depression Scale for screening post-natal depression in India: A meta-analysis. World J. Psychiatry.

[38] Zamora J., Abraira V., Muriel A., Khan K., Coomarasamy A. (2006). Meta-DiSc: A software for meta-analysis of test accuracy data. BMC Med. Res. Methodol..

[39] Yang Q., Diamond M.P., Al-Hendy A., Yang Q. (2016). The emerging role of extracellular vesicle-derived miRNAs: Implication in cancer progression and stem cell related diseases. J. Clin. Epigenet..

[40] Volinia S., Calin G., Liu C.-G., Ambs S., Cimmino A., Petrocca F., Visone R., Iorio M., Roldo C., Ferracin M. (2006). A microRNA expression signature of human solid tumors defines cancer gene targets. Proc. Natl. Acad. Sci. USA.

[41] Elnaggar G.N., El-Hifnawi N.M., Ismail A., Yahia M., Elshimy R.A. (2021). Micro RNA-148a Targets Bcl-2 in Patients with Non-Small Cell Lung Cancer. Asian Pac. J. Cancer Prev..

[42] Wan K., Tu Z., Liu Z., Cai Y., Chen Y., Ling C. (2021). Upregulated osteoprotegerin expression promotes lung cancer cell invasion by increasing miR-20a expression. Exp. Ther. Med..

[43] Xu F., Jiang L., Zhao Q., Zhang Z., Liu Y., Yang S., Yu M., Chen H., Zhang J., Zhang J. (2021). Whole-transcriptome and proteome analyses identify key differentially expressed mRNAs, miRNAs, lncRNAs and circRNAs associated with HCC. Oncogene.

[44] Lee J.C., Zhao J.-T., Gundara J., Serpell J., Bach L.A., Sidhu S. (2015). Papillary thyroid cancer–derived exosomes contain miRNA-146b and miRNA-222. J. Surg. Res..

[45] Wojtas B., Ferraz C., Stokowy T., Hauptmann S., Lange D., Dralle H., Musholt T., Jarzab B., Paschke R., Eszlinger M. (2014). Differential miRNA expression defines migration and reduced apoptosis in follicular thyroid carcinomas. Mol. Cell. Endocrinol..

[46] He H., Jazdzewski K., Li W., Liyanarachchi S., Nagy R., Volinia S., Calin G., Liu C.-G., Franssila K., Suster S. (2005). The role of microRNA genes in papillary thyroid carcinoma. Proc. Natl. Acad. Sci. USA.

[47] Tetzlaff M.T., Liu A., Xu X., Master S.R., Baldwin D.A., Tobias J.W., Livolsi V.A., Baloch Z.W. (2007). Differential Expression of miRNAs in Papillary Thyroid Carcinoma Compared to Multinodular Goiter Using Formalin Fixed Paraffin Embedded Tissues. Endocr. Pathol..

[48] Zhou G., Xiao M., Zhao L., Tang J., Zhang L. (2015). MicroRNAs as novel biomarkers for the differentiation of malignant versus benign thyroid lesions: A meta-analysis. Genet. Mol. Res..

[49] Stokowy T., Wojtas B., Fujarewicz K., Jarząb B., Eszlinger M., Paschke R. (2014). miRNAs with the Potential to Distinguish Follicular Thyroid Carcinomas from Benign Follicular Thyroid Tumors: Results of a Meta-analysis. Horm. Metab. Res..

[50] Laukiene R., Jakubkevicius V., Ambrozaityte L., Cimbalistiene L., Utkus A. (2021). Dysregulation of microRNAs as the risk factor of lymph node metastasis in papillary thyroid carcinoma: Systematic review. Endokrynol. Pol..

[51] Ito Y., Higashiyama T., Takamura Y., Miya A., Kobayashi K., Matsuzuka F., Kuma K., Miyauchi A. (2007). Risk Factors for Recurrence to the Lymph Node in Papillary Thyroid Carcinoma Patients without Preoperatively Detectable Lateral Node Metastasis: Validity of Prophylactic Modified Radical Neck Dissection. World J. Surg..

[52] Su D., Ji Z., Xue P., Guo S., Jia Q., Sun H. (2020). Long-Noncoding RNA FGD5-AS1 Enhances the Viability, Migration, and Invasion of Glioblastoma Cells by Regulating the miR-103a-3p/TPD52 Axis. Cancer Manag. Res..

[53] Jiang Y., Chen J., Yue C., Zhang H., Tong J., Li J., Chen T. (2016). The role of miR-182-5p in hepatocarcinogenesis of trichloroethylene in mice. Toxicol. Sci..

[54] Sierra-Ramirez J., Seseña-Mendez E., Godinez-Victoria M., Hernandez-Caballero M. (2021). An insight into the promoter methylation of PHF20L1 and the gene association with metastasis in breast cancer. Adv. Clin. Exp. Med..

[55] Akyay O.Z., Gov E., Kenar H., Arga K.Y., Selek A., Tarkun I., Canturk Z., Cetinarslan B., Gurbuz Y., Sahin B. (2020). Mapping the Molecular Basis and Markers of Papillary Thyroid Carcinoma Progression and Metastasis Using Global Transcriptome and microRNA Profiling. OMICS J. Integr. Biol..

[56] Cibas E.S., Ali S.Z. (2017). The 2017 Bethesda System for Reporting Thyroid Cytopathology. Thyroid.

