# Supplementary material for: Diagnostic and Prognostic Performance of Liquid Biopsy-Derived Exosomal MicroRNAs in Thyroid Cancer Patients: A Systematic Review and Meta-Analysis
Source: Cancers (Basel). 2021 Aug 26;13(17):4295. doi: 10.3390/cancers13174295 (PMC8428356; doi:10.3390/cancers13174295)
Supplement: Supplementary file 1 [file cancers-13-04295-s001.zip › cancers-1306091-supplementary.pdf]

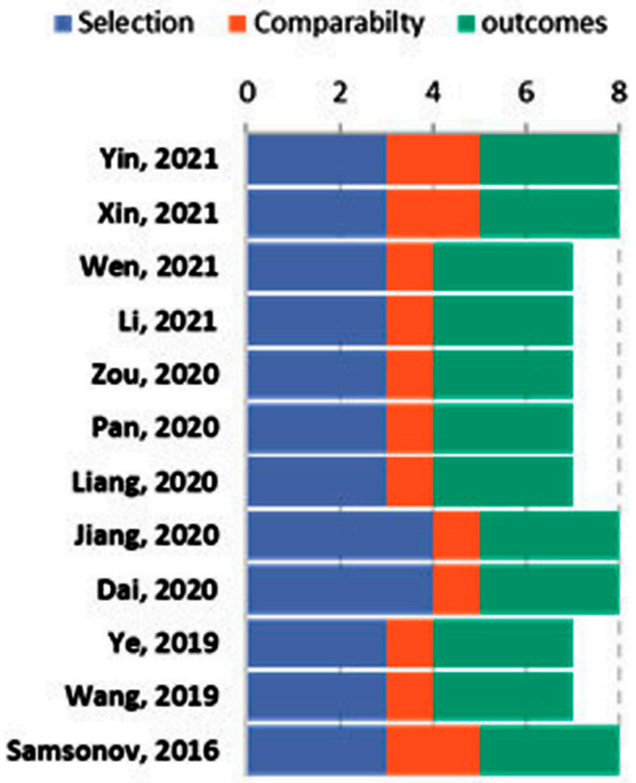

Supplementary Figure S1. Quality assessment for included studies. Evaluation using Newcastle-Ottawa Scale was carried out.

Supplementary Table S1. Major recommendations of MISEV.

**1. EV separation/isolation**
a) There is no single optimal isolation method, so choose based on the downstream applications and scientific question
b) Report all details of the method(s) for reproducibility **(1)**
**2. EV characterization**
a) General characterization. Show:
i. At least three positive protein markers of EVs, including at least one **(3)**
-transmembrane/lipid-bound protein
-cytosolic protein
ii. At least one negative protein marker **(1)**
b) Characterization of single vesicles: use two different but complementary techniques, for example:
i. electron or atomic force microscopy (and show both close-up and wide-field) **(1)**
ii. single-particle tracking **(1)**
**3. Functional studies should include:**
a) Dose-response studies **(1)**
b) Process controls to rule out the influence of serum components/other possible contaminants **(1)**
c-i.) Density gradients to show activity is intrinsic to EVs, not just associated **(1)**
**or** c-ii.) EV depletion to remove activity
**or** c-iii.) EV/cell labeling (e.g., fluorescent labeling, with careful interpretation)

MISEV: minimal information for studies of extracellular vesicles [24]. The number between brackets is the assigned score for each item that evaluates the included studies in this meta-analysis. The total score is 10.

Supplementary Table S2. Deregulated exosomal miRNAs in thyroid cancer.

miRNAs	No. cancer cases	No. controls	Assay	Log2Fold change	Cutoff (ROC)	AUC (95%CI)	Sensitivity (95%CI)	Specificity (95%CI)	Ref
**Upregulated**									
miR-25-3p	48	45 normal	qRT-PCR	0.62					[30]
miR-92a-3p	48	45 normal	qRT-PCR	0.78					[30]
miR-296-5p	48	45 normal	qRT-PCR	1.01					[30]
miR-25-3p	34	35 normal	qRT-PCR	1.28		0.623 (0.542-0.704)	64.1%	60.4%	[30]
miR-92a-3p	34	35 normal	qRT-PCR	0.83		0.702 (0.626-0.778)	67.4%	69.2%	[30]
miR-296-5p	34	35 normal	qRT-PCR	1.04		0.621 (0.54-0.70)	75%	50.5%	[30]
miR-485-3p	96	30 normal	qRT-PCR			0.866 (0.79-0.94)	80.2%	71.2%	[33]
miR-4433a-5p	96	30 normal	qRT-PCR			0.863 (0.79-0.93)	81.3%	72.9%	[33]
miR-4306	96	30 normal	qRT-PCR						[33]
miR-376a-3p	96	30 normal	qRT-PCR						[33]
miR-204-3p	96	30 normal	qRT-PCR						[33]
miR-423-5p	60	30 normal	qRT-PCR						[34]
miR-346	25	25 normal	qRT-PCR	1.08		0.834 (0.77-0.89)			[35]
miR-10a-5p	25	25 normal	qRT-PCR	1.08		0.774 (0.71-0.84)			[35]
miR-34a-5p	25	25 normal	qRT-PCR	1.33		0.760 (0.68-0.83)			[35]
miR-223-5p	35	31 normal	qRT-PCR		<0.346	0.95	90.8% (80.9-96.5)	85.7% (67.3-95.9)	[32]
miR-16-2-3p	35	31 normal	qRT-PCR		<0.301	0.938	93.8% (84.9-98.3)	89.3% (71.7-97.7)	[32]
miR-223-3p	35	31 normal	qRT-PCR		<0.536	0.861	83.1% (71.7-81.2)	78.6% (59.1-91.7)	[32]
miR-146b-5p	35	31 normal	qRT-PCR		<0.589	0.812	81.5% (69.9-90.1)	78.6% (59.1-91.7)	[32]
miR-126-3p	10	8 NG	qRT-PCR						[25]
miR-145-5p	10	8 NG	qRT-PCR						[25]
miR-31-5p	10	8 NG	qRT-PCR						[25]
miR-16-2-3p	35	30 NG	qRT-PCR	1.632	>0.120	0.687	68.6% (50.7-83.2)	66.7% (47.2-82.7)	[32]
miR-223-5p	35	30 NG	qRT-PCR	0.695	>0.157	0.679	57.1% (39.4-73.7)	80.0% (61.4-92.3)	[32]
miR-16-2-3p	16	8 NG	NGS	0.971					[32]
miR-223-3p	16	8 NG	NGS	1.042					[32]
miR-223-5p	16	8 NG	NGS	0.787					[32]
miR-433-3p	16	8 NG	NGS	0.283					[32]
miR-26a-5p	16	8 NG	NGS	0.247					[32]

miR-146b-5p	16	8 NG	NGS	0.773					[32]
miR-1307-3p	16	8 NG	NGS	0.450					[32]
miR-425-5p	16	8 NG	NGS	0.238					[32]
miR-132-5p	16	8 NG	NGS	0.283					[32]
miR-598-5p	13	7 NG	qRT-PCR						[31]
miR-3161	13	7 NG	qRT-PCR						[31]
miR-6516-5p	13	7 NG	qRT-PCR						[31]
miR-4644	13	7 NG	qRT-PCR						[31]
miR-1283	13	7 NG	qRT-PCR						[31]
miR-5189-3p	13	7 NG	qRT-PCR						[31]
miR-485-3p	96	59 NG	qRT-PCR			0.858 (0.80-0.91)	85.4%	73.3%	[33]
miR-4433a-5p	96	59 NG	qRT-PCR			0.812 (0.74-0.89)	83.3%	73.3%	[33]
miR-187-3p	17	10 NG	NGS	7.418					[33]
miR-204-3p	17	10 NG	NGS	2.613					[33]
miR-219a-5p	17	10 NG	NGS	2.13					[33]
miR-376a-3p	17	10 NG	NGS	1.644					[33]
miR-378f	17	10 NG	NGS	3.792					[33]
miR-4306	17	10 NG	NGS	7.222					[33]
miR-4433a-5p	17	10 NG	NGS	2.782					[33]
miR-485-3p	17	10 NG	NGS	6.811					[33]
miR-524-5p	17	10 NG	NGS	2.327					[33]
miR-654-5p	17	10 NG	NGS	1.376					[33]
miR-7855-5p	17	10 NG	NGS	4.512					[33]
miR-889-5p	17	10 NG	NGS	3.458					[33]
**Downregulated**									
miR-130a-3p	40	40 normal	qRT-PCR			0.828 (0.76-0.88)	88.8%	90.8%	[26]
miR-29a	119	100 normal	qRT-PCR			0.884	85.7%	78.9%	[28]
miR-34c-5p	35	31 normal	qRT-PCR		<0.339	0.880	81.5% (69.9-90.1)	85.7% (67.3-95.9)	[32]
miR-182-5p	35	31 normal	qRT-PCR		<0.396	0.908	93.9% (84.9-98.3)	82.1% (63.1-93.9)	[32]
miR-1227-3p	13	7 NG	qRT-PCR						[31]
miR-149-3p	13	7 NG	qRT-PCR						[31]
miR-210-5p	13	7 NG	qRT-PCR						[31]
miR-3662	13	7 NG	qRT-PCR						[31]
miR-187-5p	13	7 NG	qRT-PCR						[31]
miR-5010-3p	13	7 NG	qRT-PCR			0.82			[31]
miR-9-5p	16	8 NG	NGS	−0.326					[32]

miR-101-3p	16	8 NG	NGS	−0.525	[32]
miR-34c-5p	16	8 NG	NGS	−0.474	[32]

ROC: Receiver Operator Characteristics; AUC: area under the curve, CI: confidence interval; Ref: reference; NG: nodular goiter; qRT-PCR: quantitative Real-Time Reverse transcription Polymerase Chain Reaction; NGS: next-generation sequencing. The area under the curve (AUC) was used as an accurate measurement of the diagnostic marker. The larger the AUC, the better the prediction model. AUC=0.5 indicates no predictive power, whereas AUC=1 represents perfect predictive performance. Raw data with fold change was converted to log2 fold change. Within the same studies, expression values or diagnostic accuracy for different datasets for screening, training, testing, and external validation stages were reported separately.

Supplementary Table S3. Diagnostic role of combined miRNA panels in thyroid cancer.

miRNAs	No. cases	No. controls	Cutoff	AUC (95%CI)	Sensitivity (95%CI)	Specificity (95%CI)	Ref.
**Cancer compared to normal**							
miR-296-5p + miR-25-3p + miR-29a-3p	24	24		0.969 (0.927-100)			[30]
miR-296-5p + miR-25-3p + miR-29a-3p	34	35		0.727 (0.60-0.85)	65.7%	73.3%	[30]
miR-296-5p + miR-25-3p + miR-29a-3p	48	45		0.771 (0.67-0.87)	88.9%	68.9%	[30]
miR-296-5p + miR-25-3p + miR-29a-3p	18	16		0.862 (0.73-0.99)	93.3%	66.7%	[30]
miR-223-5p + miR-182-5p	35	31	>0.855	0.975	90.8% (80.9-96.5)	96.4% (81.6-99.9)	[32]
miR-146b-5p + miR-182-5p	35	31	>0.758	0.906	92.3% (82.9-97.5)	82.4% (63.1-93.9)	[32]
miR-146b-5p + miR-223-5p	35	31	>0.789	0.948	89.2% (79.1-95.6)	85.7% (67.3-95.9)	[32]
miR-146b-5p + miR-223-5p + miR-182-5p	35	31	>0.769	0.981	93.8% (84.9-98.3)	92.9% (76.5-99.1)	[32]
miR-346 + miR-10a-5p + miR-34a-5p	120	114		0.825 (0.78-0.88)			[35]
miR-346 + miR-10a-5p + miR-34a-5p	30	30		0.926 (0.86-0.99)			[35]
miR-346 + miR-10a-5p + miR-34a-5p	57	54		0.811 (0.73-0.88)			[35]
miR-346 + miR-10a-5p + miR-34a-5p	33	30		0.816 (0.71-0.92)			[35]
**Cancer compared to NG**							
miR-346 + miR-10a-5p + miR-34a-5p	29	29		0.887 (0.81-0.97)			[35]
miR-16-2-3p + miR-223-5p	35	30	<0.922	0.704	54.3% (36.6-71.2)	90.0% (73.5-97.9)	[32]
miR-16-2-3p + miR-223-5p + miR-34c-5p	35	30	<0.931	0.72	60.0%	86.7%	[32]
miR-16-2-3p + miR-223-5p + miR-34c-5p + miR-101-3p	35	30	<0.946	0.735	71.4% (53.7-85.4)	73.3% (54.1-87.7)	[32]
miR-223-5p + miR-34c-5p + miR-101-3p + miR-146b-5p	35	30	<0.946	0.730	74.3% (56.7-87.5)	66.7% (47.2-82.7)	[32]

qRT-PCR was performed in all studies. NG: nodular goiter.

Supplementary Table S4. Exosomal miRNAs associated with lymph node metastasis in thyroid cancer patients.

miRNAs	No. LNM	No. LNM	Expression	FC	*p*-value	Cutoff	AUC (95%CI)	Sensitivity	Specificity	Ref.
miR-21-5p	49	15	High		0.021					[21]
miR-204-5p	49	15	High		0.015					[21]
miR-221-3p	49	15	High		0.036					[21]
miR-146b-5p	49	15	High		0.008		0.811	76.6%	86.7%	[21]
miR-222-3p	49	15	High		0.007		0.834	78.7%	80.0%	[21]
miR-146b-5p + miR-222-3p	49	15	High				0.895	85.1%	80.0%	[21]
miR-485-3p	52	44	High				0.805			[33]
miR-182-5p	8	8	High	0.9659	0.005	0.428	0.641 (0.345, 0.937)	87.5%	50%	[32]
miR-26b-5p	8	8	High	0.5895	0.048	0.223	0.828 (0.622, 1.035)	100%	50%	[32]
miR-126-3p	8	8	High	0.4947	0.030	0.373	0.688 (0.402, 0.973)	100%	50%	[32]
miR-542-3p	8	8	High	0.4778	0.045	0.680	0.969 (0.896, 1.042)	87.5%	100%	[32]
miR-32-5p	8	8	High	0.4653	0.034	0.232	0.953 (0.862, 1.044)	100%	75%	[32]
miR-363-3p	8	8	High	0.4385	0.021	0.688	0.828 (0.612, 1.045)	75%	87.5%	[32]
miR-130a-3p			Low							[26]
miR-148a-3p			Low							[29]
miR-1912	8	8	Low	−1.3324	0.023	0.517	0.672 (0.38, 0.964)	100%	50%	[32]
miR-323a-5p	8	8	Low	−1.1819	0.018	0.621	0.859 (0.638, 1.081)	87.5%	87.5%	[32]
miR-543	8	8	Low	−1.1389	0.023	0.642	0.859 (0.638, 1.081)	87.5%	87.5%	[32]
miR-381-3p	8	8	Low	−1.0414	0.006	0.541	0.766 (0.504, 1.027)	100%	62.5%	[32]
miR-128-3p	8	8	Low	−0.9953	0.019	0.578	0.781 (0.534, 1.028)	100%	62.5%	[32]
miR-139-5p	8	8	Low	−0.6265	0.040	0.367	0.828 (0.622, 1.035)	100%	50%	[32]
miR-885-3p	8	8	Low	−0.6164	0.036	0.401	0.797 (0.561, 1.032)	100%	62.5%	[32]
miR-409-5p	8	8	Low	−0.5834	0.024	0.405	0.594 (0.292, 0.896)	87.5%	37.5%	[32]
miR-28-5p	8	8	Low	−0.4521	0.009	0.559	0.562 (0.248, 0.877)	37.5%	100%	[32]
miR-151a-5p	8	8	Low	−0.4198	0.047	0.504	0.688 (0.395, 0.98)	75%	75%	[32]
miR-490-3p	8	8	Low	−0.3696	0.036	0.505	0.547 (0.227, 0.867)	75%	62.5%	[32]

LNM: lymph node metastasis; FC: log2 fold change; AUC: area under the curve.

Supplementary Table S5. List of target genes of KEGG pathways enriched in thyroid cancer exosomal miRNA meta-signature.

KEGG pathway	Hits/Pop Hits	Target genes
hsa04068 FoxO signaling pathway	10/131	SGK3; BCL6; TGFBR1; SIRT1; BCL2L11; CCND2; FOXO1; MAPK1; MAP2K1; SMAD4
hsa04218 Cellular senescence	12/160	TSC1; E2F5; TGFBR1; SIRT1; ETS1; CDK6; E2F3; CCND2; FOXO1; MAPK1; MAP2K1; RRAS
hsa05200 Pathways in cancer	24/531	ESR1; MITF; IL6R; BCL2; TPM3; LEF1; TGFBR1; ETS1; CDK6; FGFR1; BCL2L11; E2F3; GNA13; PDGFRA; CCND2; FOXO1; GNAQ; MAPK1; MAP2K1; TRAF6; SMAD4; CCDC6; APPL1; PLCB1
hsa04151 PI3K-Akt signaling pathway	13/354	TSC1; IL6R; BDNF; BCL2L11; CREB3L2; PHLPP2; RBL2; PDGFRA; ITGA5; ITGB8; NRAS; EFNA1; CCNE2
hsa05215 Prostate cancer	10/97	BCL2; LEF1; ERG; FGFR1; E2F3; CREB3L2; PDGFRA; FOXO1; MAPK1; MAP2K1
hsa05210 Colorectal cancer	8/86	BCL2; LEF1; TGFBR1; BCL2L11; MAPK1; MAP2K1; SMAD4; APPL1
hsa05218 Melanoma	7/72	MITF; CDK6; FGFR1; E2F3; PDGFRA; MAPK1; MAP2K1
hsa04919 Thyroid hormone signaling pathway	7/137	THRB; RCAN1; EP300; ATP2A2; NRAS; PLCB1; NOTCH1
hsa05206 MicroRNAs in cancer	12/310	BCL2; FOXP1; SIRT1; CDK6; BMPR2; BCL2L11; E2F3; RDX; PDGFRA; CCND2; MAPK1; MAP2K1, TGFB2; SOX4
hsa01521 EGFR tyrosine kinase inhibitor resistance	7/79	IL6R; BCL2; BCL2L11; AXL; PDGFRA; MAPK1; MAP2K1
hsa05216 Thyroid cancer	5/37	TPM3; LEF1; MAPK1; MAP2K1; CCDC6
hsa04520 Adherens junction	6/71	LEF1; TGFBR1; FGFR1; ACTG1; MAPK1; SMAD4
hsa04211 Longevity regulating pathway	6/89	TSC1; SIRT1; CREB3L2; SESN3; FOXO1; APPL1
hsa04115 p53 signaling pathway	5/72	BCL2; CDK6; SESN3; ZMAT3; CCND2
hsa04140 Autophagy	7/137	TSC1; BCL2; MAPK1; MAP2K1; TRAF6; RRAS; ATG2B
hsa04810 Regulation of actin cytoskeleton	9/213	FGFR1; ACTG1; RDX; GNA13; PDGFRA; TIAM1; MAPK1; MAP2K1; RRAS
hsa04550 Signaling pathways regulating pluripotency of stem cells	7/142	FGFR1; REST; BMPR2; PCGF3; MAPK1; MAP2K1; SMAD4
